# LINE-1 ORF1p as a candidate biomarker in high grade serous ovarian carcinoma

**DOI:** 10.1038/s41598-023-28840-5

**Published:** 2023-01-27

**Authors:** Sho Sato, Michael Gillette, Pamela R. de Santiago, Eric Kuhn, Michael Burgess, Kristen Doucette, Yi Feng, Carlos Mendez-Dorantes, Paul J. Ippoliti, Sara Hobday, Marilyn A. Mitchell, Kai Doberstein, Stefan M. Gysler, Michelle S. Hirsch, Lauren Schwartz, Michael J. Birrer, Steven J. Skates, Kathleen H. Burns, Steven A. Carr, Ronny Drapkin

**Affiliations:** 1 Penn Ovarian Cancer Research Center, University of Pennsylvania, Perelman School of Medicine, Philadelphia, PA 19104 USA; 2 The Broad Institute of MIT and Harvard, Cambridge, MA 02142 USA; 3 Division of Pulmonary and Critical Care Medicine, Massachusetts General Hospital, Boston, MA 02114 USA; 4 Department of Pathology, Brigham and Women’s Hospital, Boston, MA 02115 USA; 5 Department of Pathology and Laboratory Medicine, Hospital of the University of Pennsylvania, Philadelphia, PA 19104 USA; 6 Harvard Medical School, Boston, MA 02115 USA; 7 Department of Medicine, University of Alabama at Birmingham, Birmingham, AL 35233 USA; 8 Biostatistics and Computational Biology, Massachusetts General Hospital, Boston, MA USA; 9 Department of Oncologic Pathology, Dana-Farber Cancer Institute, Boston, MA USA; 10 Basser Center for BRCA, Abramson Cancer Center, University of Pennsylvania, Perelman School of Medicine, Philadelphia, PA 19104 USA

**Keywords:** Biochemistry, Biological techniques, Cancer, Cell biology, Molecular biology, Oncology, Pathogenesis

## Abstract

Long interspersed element 1 (LINE-1) open reading frame 1 protein (ORF1p) expression is a common feature of many cancer types, including high-grade serous ovarian carcinoma (HGSOC). Here, we report that ORF1p is not only expressed but also released by ovarian cancer and primary tumor cells. Immuno-multiple reaction monitoring-mass spectrometry assays showed that released ORF1p is confidently detectable in conditioned media, ascites, and patients’ plasma, implicating ORF1p as a potential biomarker. Interestingly, ORF1p expression is detectable in fallopian tube (FT) epithelial precursors of HGSOC but not in benign FT, suggesting that ORF1p expression in an early event in HGSOC development. Finally, treatment of FT cells with DNA methyltransferase inhibitors led to robust expression and release of ORF1p, validating the regulatory role of DNA methylation in LINE-1 repression in non-tumorigenic tissue.

## Introduction

Ovarian cancer remains a leading cause of cancer-related mortality in developed countries, accounting for approximately 180,000 deaths worldwide annually^1^. In the United States alone, the American Cancer Society estimates 19,880 new cases and over 12,810 deaths occurred from ovarian cancer in 2022^2,3^. Ovarian cancer is a heterogeneous disease and the most common subtype is high-grade serous ovarian carcinoma (HGSOC)^4^. Most patients with HGSOC present to care with advanced disease, at which point prolonged remissions after primary therapy are rare, and recurrences are marked by increasing chemoresistance. Thus, there is a dire need for improved strategies for the early detection of ovarian cancer to reduce mortality^5,6^. Unfortunately, an adequately sensitive and specific screening test that improves survival has yet to be developed. Currently, the best available tool for evaluation of ovarian disease is transvaginal ultrasound (TVS). However, multiple large-scale studies have failed to demonstrate adequate sensitivity and specificity for TVS to warrant its use as a screening tool^7,8^. While CA-125 and HE4 are well-characterized biomarkers in ovarian cancer^9–13^, their clinical application is currently restricted to analysis of therapeutic efficacy and detection of disease relapse^14^. Recent findings from the United Kingdom Collaborative Trial of Ovarian Cancer Screening (UKCTOCS) study suggest that multimodal screening with serum CA-125 interpreted using the Risk of Ovarian Cancer Algorithm (ROCA), transvaginal ultrasound, and clinical assessment can lead to a shift to earlier-stage detection and treatment^15^. Unfortunately, long-term follow-up in the UKCTOCS study showed that the reduction in stage III or IV incidence seen with multimodal screening did not translate into lives saved^16^. It is important to note that approximately 20% of ovarian cancers do not produce CA-125 and would be missed by an approach dependent solely on this biomarker^17^. Together, these findings reinforce the need for the identification of broadly applicable biomarkers that stem from a more complete understanding of disease biology.

With the evolution of proteomic technologies, it is possible to undertake a systematic characterization of proteins that are released to the interstitial space by resident cell types in the tumor microenvironment. Tissue interstitial fluid (TIF) is comprised of the fluid between blood vessels and surrounding tissue cells, constitutes 16% of the weight of the human body, and represents a novel and highly promising source of biomarkers^18^. We recently completed a proteome-wide analysis of TIF from normal fallopian tube (FT) epithelium and matched HGSOC (Gillette et al., manuscript in preparation). Among the proteins most differentially detected in the TIF of HGSOC is the long-interspersed element-1 (LINE-1) retrotransposable element ORF1 protein (ORF1p).

Transposable elements (TEs) can be separated into two major classes: DNA transposons and retrotransposons. Retrotransposons are by far the most abundant TEs in the human genome and self-propagate via RNA intermediates that are reverse-transcribed and inserted into new genomic locations^19^. Retrotransposons can be further subdivided into two groups, distinguished by the presence or absence of long terminal repeats (LTRs). The vast majority of human TEs reflect the present and past activity of non-LTR retrotransposons, typified by LINE-1^20,21^. LINE-1 elements are the most abundant and only active protein-coding retrotransposons, accounting for approximately 17% of the human genome. Roughly, 500,000 truncated copies and 6000 full-length LINE-1 copies are present in the human genome^22^. Transcription is driven by a CpG dinucleotide-rich internal RNA polymerase II promoter^21,22^ but expression in adult human cells is usually suppressed by DNA methylation^20^. Interestingly, loss of LINE-1 DNA methylation is a common phenotype found in ovarian and other cancer types^19^.

LINE-1 contains two open reading frames (ORFs): ORF1 encodes an RNA binding protein (ORF1p), and ORF2 encodes a protein with reverse transcriptase and endonuclease activities (ORF2p)^21,23^. ORF1p aberrant expression has been documented in a number of studies across epithelial cancers, including ovarian^24–31^, and makes ORF1p a promising cancer biomarker. Despite the high levels of ORF1p found in cancer cells, ORF2p expression has been never shown using standard methods for protein detection^32^.

In the present study, we show that ORF1p is not only expressed but also released by HGSOC and primary tumor cell lines. We developed immuno-affinity enrichment at the peptide level assays (Immuno-MRM, iMRM^33–40^) combined with targeted mass spectrometry to detect secreted ORF1p in conditioned media, ascites, and patient plasma samples. We also show that the expression of ORF1p is an early event in the development of HGSOC, as ORF1p staining is robustly found in STIC lesions samples. Moreover, ORF1p was found in incidental STIC lesions from risk-reducing surgeries and with no invasive disease, validating that the transition from normal FT epithelium to HGSOC precursor lesions is marked by the acquisition of ORF1p expression that is retained in advanced HGSOC. Interestingly, we found that DNA demethylating agents trigger ORF1p expression and release by FT cells, thereby experimentally validating DNA methylation as a necessary mechanism to restrain ORF1p expression in benign FT cells.

## Methods

### Case selection

The cases for this study were obtained from the Departments of Pathology at Brigham and Women’s Hospital and the Hospital of the University of Pennsylvania. Formalin-fixed paraffin embedded (FFPE) blocks of fallopian tube tissues were cut from 30 cases whose original pathology reports indicated the presence of HGSOC. Among those cases, 18 included the diagnosis of STIC and 25 had benign FT epithelium. We also obtained 12 cases of benign FT from patients who had their tubes removed for reasons unrelated to cancer or cancer-risk and six cases of incidental STIC (In-situ carcinoma only) lesions identified at risk-reducing surgery for *BRCA1/2* mutations. These hematoxylin and eosin (H&E) slides were reviewed by three pathologists (MSH, LS, RD) to confirm the presence of STICs and possibly invasive carcinoma in the deeper tissue sections. The plasma samples used in this study were obtained from the repository of Dr. Steven Skates at the Massachusetts General Hospital. Seventy-two plasma samples from patients with advanced stage (III and IV) (Supplementary Table 1) papillary serous ovarian carcinoma, and 37 control plasma samples were analyzed.

### Immunohistochemistry (IHC)

Immunohistochemical staining was performed using Envision Plus/Horseradish Peroxidase system (DAKO). FFPE tissue sections were deparaffinized, rehydrated, and incubated in hydrogen peroxide solution for 30 min to block endogenous peroxidase activity. Antigen retrieval was carried out at 100 °C treatment in citrate buffer (pH 6.0) for 20 min. Sections were incubated with primary antibody overnight at 4 °C. The secondary antibody was applied for 30 min, followed by 3,3′-Diaminobenzidine (DAB) for 5 min. All H&E and IHC images were captured with the Leica BioSystems (Buffalo Grove, IL) Aperio CS2 slide scanner.

### ORF1p IHC scoring

We used monoclonal anti-LORF1 antibody (clone 4H1; Millipore) to investigate ORF1p expression. ORF1p staining was scored by two gynecologic pathologists (LES and MSH) using the following 4-tiered scale: 0 (all cells negative), 1 + (scattered rare cells ≤ 10% positive cells), 2 + (focal or multifocal staining = 10–75% positive cells), or 3 + (diffuse staining ≥ 75% positive cells). All stains and scores were reviewed by a third pathologist (RD) and dichotomized into two groups: scores of 0 and 1 + were defined as “ORF1p negative”, while scores of 2+ and 3+ were categorized as “ORF1p positive”.

### Immunofluorescence microscopy

Cells were grown overnight on glass cover slips. Cells were fixed with 4% paraformaldehyde/PBS for 20 min at room temperature. Cells were blocked with 3% BSA in 1X PBS and incubated with primary antibody overnight at 4 °C. The secondary antibody was incubated for 0.5 h at room temperature. Detection was performed using secondary antibodies conjugated to Alexa 488 Fluor Dyes (Molecular Probes; Thermo Fisher Scientific). Cover slips were mounted onto glass slides using DAPI-containing medium. Cells were analyzed by microscopy using a Nikon E400 microscope.

### Cell lines

Eight ovarian carcinoma cell lines (KURAMOCHI, OVCAR-3, OVCAR-4, OVCAR-8, OVKATE, COV318, OVSAHO and CaOV3) were used in this study. All ovarian cancer cell lines, except COV318 and CaOV3, were cultured in RPMI 1640 (Thermo Fisher Scientific) supplemented with 10% fetal bovine serum (FBS; Atlanta Biologicals). COV318 and CaOV3 were cultured in Dulbecco’s Modified Eagle’s Medium (DMEM; Thermo Fisher Scientific) with 10% fetal bovine serum. All cell lines were authenticated using Short Tandem Repeat (STR) profiling (IDEXX, Columbus, MO) in 2022 and tested to be free of *Mycoplasma* using the Cambrex MycoAlert assay (University of Pennsylvania Perelman School of Medicine Cell Center). The establishment of the fallopian tube cell lines (FT189, FT194, FT237, FT240 and FT246) was previously described^41,42^. FT cells were cultured in DMEM/Ham’s F-12 1:1 (Thermo Fisher Scientific) supplemented with 2% Ultroser G serum substitute (Pall Life Sciences). Primary HGSOC cells (DF cell lines) were isolated as previously described^43^. DF cells were cultured in Renaissance Essential Tumor Medium (RETM; Cellaria Biosciences) supplemented with 5% heat-inactivated FBS. All cells were grown at 37 °C and a 5% CO_2_-containing atmosphere (see Supplementary Table 2).

### Decitabine and SGI-110 treatments

Cultured FT cell lines were treated with Decitabine (TOCRIS, Cat. #2624) or SGI-110 (Guadecitabine) (Adooq Bioscience, Cat. #A12744) at a concentration of 5 μM (in DMSO) for 3, 5 or 7 days. As control, cells were treated with vehicle alone.

### Conditioned medium

All cells were grown to 80% confluence. The media was then change to media without FBS (ovarian cancer carcinoma cell lines) or Ultroser G serum substitute (Pall Life Sciences) (FT cell lines) and the cells were cultured for an additional 72 h. The conditioned medium was then cleared by centrifugation and concentrated using a Millipore Amicon Ultra-15 centrifugal filter (Millipore Sigma). Protein content of conditioned medium was quantified using the Pierce BCA kit protocol (Thermo Fisher Scientific) and western bot was performed as described below.

### Western blot

Whole cell lysates were prepared using M-PER buffer (Thermo Fisher Scientific). Protein content of whole cell lysate was quantified using the Pierce BCA kit protocol (Thermo Fisher Scientific). Proteins (20–30 µg) were separated on a 4–20% gradient SDS-PAGE before being transferred to a PVDF membrane using the Turbo Blot system (Bio-Rad). Membranes were incubated with primary antibodies overnight at 4 °C (see Supplementary Table 3). After washing, membranes were incubated with HRP-conjugated secondary antibodies for 1 h at room temperature. Proteins were detected using Clarity Chemiluminescent HRP Antibody Detection Reagent (Bio-Rad) and visualized with a Chemi-Doc imaging system (Bio-Rad). The uncropped blots for all the figures can be found in Supplementary Figs. 1–4.

### LINE-1 methylation assay

LINE-1 methylation was assessed using the Global DNA Methylation—LINE-1 Kit (Active Motif, Cat. #55017) following the manufacturer’s recommendations. LINE-1 Kit is an ELISA-based assay for the detection and quantification of 5-methylcytosine levels in human genomic DNA. Briefly, FT cell lines were treated with decitabine or DMSO as previously described. Following, genomic DNA (gDNA) was extracted using the DNeasy Blood & Tissue Kit (Qiagen, Cat. #69504). One μg of gDNA of each sample was digested overnight with MseI enzyme (10 U/μL) at 37 °C. 100 ng of digested gDNA was hybridized with LINE-1 probe in a thermal cycler (98 °C for 10 min, 68 °C for 1 h followed by a quick ramp to 25 °C). LINE-1 probe is a 5’ biotinylated oligo designed to hybridize to a 290 bp region of the LINE-1 repeat element. This region contains 88 cytosine residues, of which 12 are in a CpG context. Reactions were prepared in technical triplicate. PCR samples were transferred to a streptavidin-coated plate and incubated for 1 h at room temperature with mild agitation. Then, 1:100 dilution of 5-methylcytosine monoclonal antibody was incubated for 1 h at room temperature followed by 1 h incubation of HRP-conjugated secondary antibody. Developing solution was added and incubated for 3 min until the addition of Stop solution. Finally, the plate was read at 450 nm. Methylated and non-methylated DNA standard samples were prepared in parallel with decitabine-treated samples.

### Immuno multiple reaction monitoring-mass spectrometry (iMRM-MS)

#### Sample digestion and desalt

##### Conditioned media

Conditioned media from ovarian cancer or control fallopian epithelial cells were digested with porcine trypsin following our previously described urea protocol^33^. Briefly, 36 mg Urea (Sigma-Aldrich) was added to 100 μL of conditioned media to a final concentration of 6 M. The pH of the solution was adjusted to 8.0 as necessary with 1 M Tris pH 8.0. Proteins were reduced with 6 μL 0.5 M tris(2-carboxyethyl) phosphine (TCEP) (Bio-Rad) and incubated 30 min at 37 °C. Samples were cooled to room temperature and alkylated by addition of 12 μL of 0.5 M iodoacetamide (IAA) (Sigma-Aldrich) freshly prepared from powder prior to incubation in the absence of direct light for 30 min at room temperature. Urea concentration was diluted to less than 2 M with 400 μL 0.2 M Tris HCl pH 8.1, 2 ug trypsin (Promega) was added (avg. 1:50 E:S), and samples were incubated on a thermomixer (Eppendorf) for 16 h at 37 °C and 800 RPM. After 16 h, 2 μg fresh trypsin was added and incubated 2 h at 37 °C and 800 RPM. After 2 h, 20 μL of neat formic acid was added to quench the reaction (final pH < 2.5). Digested samples were desalted separately using 10 mg Oasis® HLB extraction cartridges mounted to a vacuum manifold (Waters). Prior to sample loading, cartridges were wetted with 1 mL 90% acetonitrile/0.1% formic acid and equilibrated with 1 mL 0.1% formic acid. After each sample was loaded, cartridges were washed with 3 mL 0.1% formic acid and peptides were eluted into a fresh 1.5 mL polytube using two additions of 0.3 mL 40% acetonitrile/0.1% formic acid. Samples were dried by vacuum centrifugation and stored dry at − 80 °C until use.

##### Ascites fluid

Ascites fluid collected from ovarian cancer patients was digested with lys’C and porcine trypsin following our previously described urea protocol^34^. Briefly, 60 μL of 9 M urea (Sigma-Aldrich) was added to 30 μL ascites fluid (avg. protein concentration 33.7 mg/mL by BCA) to a final concentration of 6 M. The pH of the solution was adjusted to 8.0 as necessary with 1 M Tris pH 8.0. Proteins were reduced with 6 μL 0.5 M tris(2-carboxyethyl) phosphine (TCEP) (Bio-Rad) and incubated 30 min at 37 °C. Samples were cooled to room temperature and alkylated by addition of 12 μL of 0.5 M iodoacetamide (IAA) (Sigma-Aldrich) freshly prepared from powder prior to incubation in the absence of direct light for 30 min at room temperature. Urea concentration was diluted to 1.5 M with 300 μL 0.2 M Tris HCl pH 8.1. Lysyl Endopeptidase (lys-C) (Wako) was dissolved in 50 mM acetic acid to a concentration of 0.5 mg/mL and 40 μL was added to each sample (avg. 1:50 E:S). Samples were incubated on a thermomixer (Eppendorf) for 2 h at 37 °C and 800 RPM. After 2 h, 10 μg trypsin (Promega) was added (avg. 1:100 E:S) and samples were incubated 16 h at 37 °C and 800 RPM. After 16 h, 10 μg fresh trypsin was added and incubated 2 h at 37 °C and 800 RPM. Twenty microliters of neat formic acid were added to quench the reaction (pH < 2.5). Heavy peptide standards (75 fmol each) were added to each digest well and samples were desalted using a 30 mg Oasis® HLB extraction plate (Waters) mounted onto a positive pressure manifold (Waters). Wells were washed with 1.5 mL 80% acetonitrile/0.1% formic acid and equilibrated with 2 mL 0.1% formic acid applying 15 psi. An additional 0.2 mL 0.1% formic acid was added to the wet cartridge prior to transferring the samples using a multichannel pipet. After the samples were loaded, cartridges were washed by positive pressure (9 psi) with 3 mL 0.1% formic acid. After washing, digested plasma peptides were eluted with two volumes of 0.5 mL of 50% acetonitrile/0.1% formic acid (6 psi). Elution plate was covered with BioExcell® film (World Wide Medical Products), frozen and dried by vacuum centrifugation, then sealed with aluminum foil and stored at − 80 °C until use.

##### Plasma

Forty microliters of plasma per ovarian cancer patient was manually dispensed into 96 deep well plates in triplicate and digested with lys-C and trypsin using our urea protocol adapted for automation as previously described^34^. Briefly, 100 μL 9 M Urea and 25 μL 0.25 M TCEP were added to quadrants 1 and 2 of a 384-well plate (Greiner) for each corresponding well of a 96-well plate containing sample (Supplementary Fig. 5A) and placed in position 7 on a Bravo LT robot (Agilent) (Supplementary Fig. 6). Sample plate, reagent plates, pipet tips and solvent plates were loaded onto Bravo LT as shown in Supplementary Fig. 6. Bravo LT was covered with a custom black shroud to minimize light penetrance and the Bravo digestion program was started. Eighty microliters 9 M Urea and 15 μL 0.25 M tris(2-carboxyethyl) phosphine (TCEP) (Bio-Rad) were added to each sample and the sample plate was moved onto the temperature-controlled shaker in position 4 (Supplementary Fig. 6) and incubated 30 min at 37 °C and 800 RPM. Iodoacetamide (IAA) (Sigma-Aldrich) was dissolved in 0.2 M Tris HCl pH 8.1 to a final concentration of 0.5 M and 50 μL was added into quadrant 3 of the 384-well plate in position 7 on the Bravo deck (Supplementary Fig. 6). After 30 min TCEP protein denaturation, Bravo robot moved the sample plate back to position 5 and added 20 μL of 0.5 M IAA into each sample before incubating 30 min without mixing in the dark. Lysyl Endopeptidase (lys-C) (Wako) was dissolved in 50 mM acetic acid to a concentration of 0.5 mg/mL and 100 μL was added to quadrant 1 of 384-well plate 2 (Supplementary Fig. 5B). One hundred microliters porcine trypsin (Promega) formulated in 50 mM acetic acid at 0.5 mg/mL was added to quadrant 2 of plate 2 (Supplementary Fig. 5B) and placed into position 6 on Bravo LT (Supplementary Fig. 6). After 30 min IAA protein alkylation, 300 μL of 0.2 M Tris HCl pH 8.1 and 100 μL of lys-C (E:S 1:50) were added. The sample plate was manually transferred to an off-line thermomixer (VWR) and incubated 2 h at 37 °C and 800 RPM. After lys-C digestion, sample plate was placed back into position 5 on Bravo deck (Supplementary Fig. 6) and 48 μL of trypsin was aspirated into each sample well (E:S 1:100). The sample plate was manually transferred to an off-line thermomixer (VWR) and incubated 2 h at 37 °C and 800 RPM. After 2 h, a second trypsin addition of 48 μL was aspirated into the samples. The Bravo method was then paused, and the plate was covered with plastic seal and incubated 16 h at 37 °C and 800 RPM on off-line thermomixer. After 16 h, 90 μL of 10% formic acid was dispensed into each sample to quench the enzymatic activity (1% final concentration). Heavy peptide standards (150 fmol each) were added to each digest well and desalted using a 30 mg Oasis® HLB extraction plate (Waters) mounted onto a positive pressure manifold (Waters). Wells were washed with 1.5 mL 80% acetonitrile/0.1% formic acid and equilibrated with 2 mL 0.1% formic acid applying 15 psi. An additional 0.2 mL 0.1% formic acid was added to the wet cartridge prior to transferring the samples using a multichannel pipet. After the samples were loaded, cartridges were washed by positive pressure (9 psi) with 3 mL 0.1% formic acid. After washing, digested plasma peptides were eluted with two volumes of 0.5 mL of 50% acetonitrile/0.1% formic acid (6 psi). Elution plate was covered with BioExcell® film (World Wide Medical Products), frozen and dried by vacuum centrifugation, then sealed with aluminum foil and stored at − 80 °C until use.

##### Generation of anti-peptide polyclonal antibodies and heavy isotope standard peptides

Four peptides unique to the LINE-1 ORF1p/L1RE1 gene product (LORF1, Uniprot Q9UN81, LORF1_HUMAN) were selected as immunogens for antibody generation: LTADLSAETLQAR, LSFISEGEIK, LIGVPESDVENGTK, and NEQSLQEIWDYVK. Rabbit polyclonal antibodies were generated in New Zealand white rabbits following a standard 77-days protocol (New England Peptide) as previously described with modifications^35^. In brief, peptides were synthesized to 85% purity with an additional cysteine on the N-terminus and conjugated to KLH for immunization. Peptides were combined and two rabbits were immunized in descending doses over 70 days. Antisera titers of the final bleeds were measured by peptide ELISA. Two peptides with the highest titer, LSFISEGEIK and LIGVPESDVENGTK, were serially purified from a pool of final bleeds by affinity chromatography using a Sulfolink column (Thermo Fisher Scientific) bound with the immunizing peptide. In short, the sera was bound to the column containing the peptide with the lowest titer; then the flow through, expected to contain antibodies specific to subsequent higher titer peptides, was bound to the column containing the peptide with the next highest titer in order to maximize yield for each antibody. After the antiserum was bound, an extended wash (> 100 CV) was used to reduce latent passenger peptide prior to elution with glycine buffer pH 2.5. Purified antibodies were dialyzed into 25% glycerol/1X PBS/0.1% NaN3 and stored at − 20 °C until use.

##### Evaluation of passenger peptide and antibody crosslinking to Protein G beads

Twenty micrograms of each antibody was incubated with Protein G magnetic beads (Thermo Fisher Scientific) at 4 °C overnight using a 2:1 bead volume to μg antibody ratio. After washing beads with 1X PBS/0.03% CHAPS, half of the antibody beads were crosslinked using 20 mM DMP as previously described^37^. Antibody capture efficiency and passenger peptide determination were performed by adding heavy peptides to digested control plasma or buffer and enriching using antibodies with and without crosslinking (see “Automated Peptide Immunoaffinity Enrichment on KingFisher” Section). Extracted ion chromatograms of heavy peptides were used to compare capture efficiency while the relative abundance of light peptide signal was used to estimate the percentage of passenger peptide in the antibody. Corresponding “heavy” peptides containing a stable isotope-labeled lysine at the C-terminus were synthesized, purified to greater than 95%, formulated in 30% acetonitrile/0.1% formic acid and quantified by amino acid analysis (New England Peptide). Heavy peptides were analyzed by LC-MRM-MS as a mixture for “light” (endogenous) and “heavy” (standard) versions of the peptide to determine the relative amount of unlabeled peptide in the heavy peptide standard (see “NanoLC-MRM-MS Analysis” Section).

##### Automated peptide immunoaffinity enrichment on kingfisher

Dried digested samples from 100 μL of conditioned media, 30 μL ascites fluid or 20 μL plasma were resuspended in 200 μL 1 × PBS/150 mM Tris pH 8.0/0.03% CHAPS and vortex-mixed briefly at room temperature before transfer to a 250 μL KingFisher well plate (except plasma samples, which were dried directly into the well plate). Peptides were extracted using magnetic beads on KingFisherTM magnetic bead handler by immunoaffinity enrichment (IAE) as described previously^33^. In brief, a mixture of antibodies containing an optimized amount of Ab per IAE (e.g. 0.5 μg, 1 μg, or 2 μg) were bound onto 1 μm Protein G magnetic beads (Thermo Fisher Scientific) by tumble mixing (Labquake® (Thermo Fisher Scientific)) using 2 μL beads/1 μg antibody overnight at 4 °C. Antibody beads were washed twice with 1 × PBS/0.03% CHAPS, resuspended in an equivalent volume 1 × PBS/0.03% CHAPS and added to each well. The plate was sealed with an aluminum foil adhesive and gently tumble mixed overnight at 4 °C. After incubation, the plate was transferred onto a KingFisherTM magnetic bead processor (Thermo Fisher Scientific) equipped with a PCR magnet head. Beads were washed twice with 250 μL 1 × PBS/0.03% CHAPS for 1.5 min and once with 0.1 × PBS/0.03% CHAPS for 1.5 min. After washing, beads were transferred into a 100 μL PCR plate containing 50 μL of 3% acetonitrile/5% acetic acid to elute the bound material. After elution, beads were collected in a plate containing 200 μL 1X PBS/0.03% CHAPS/0.1% sodium azide.

##### Automated desalting of immunoaffinity enriched samples

Antibody enriched samples were desalted using AssayMAP Bravo Reverse Phase S (RPS) cartridges^34^. Antibody bead eluates in a 96 well Bio-Rad PCR plate were placed on Bravo in position 6. AssayMAP RPS cartridges and solvents were placed on Bravo as outlined in Supplementary Fig. 6. RPS cartridges were primed with 50 µL 90% acetonitrile/0.01% formic acid at 300 μL/min and equilibrated with 50 μL 0.1% formic acid at 25 μL/min. Samples were then loaded onto cartridges at 2 μL/min. Cartridges were washed twice with 50 μL of 0.1% formic acid and eluted with 50 μL 40% acetonitrile/0.01% formic acid at 5 μL/min. Eluates were dried and resuspended in 3% acetonitrile/5% acetic acid prior to LC-MRM-MS analysis.

##### NanoLC-MRM-MS analysis^34^

Antibody-enriched desalted samples from conditioned media, ascites and plasma were analyzed on a TSQ Quantiva triple quadrupole mass spectrometer installed with a Nanospray Flex source (Thermo Fisher Scientific) and Easy-nLC 1000 system. Ion source was set to positive ion mode with capillary temperature of 300C, spray voltage of 2000 and sweep gas set to 0. Easy-nLC 1000 system was primed with mobile phase A (3% acetonitrile/0.1% formic acid), mobile phase B (90% acetonitrile/0.1% formic acid). Samples were injected (4 μL, 40% of enriched sample) onto a 0.075 mm ID PicoFrit (New Objective) column pulled to a 10 μm emitter and custom-packed to 20 cm with 1.9 μm 200 Å C18-AQ Reprosil beads (Dr. Maisch). The LC gradient was 5% B for 3 min, 5% B to 40% B in 50 min, 40% B to 90% B in 2.3 min. Three transitions were monitored per peptide by scheduled MRM using an 8-min RT window and a 1.5 s cycle time. Collision energies were optimized over 4 steps, 2.5 V per step in smaller unscheduled batches of less than 500 transitions per batch.

Extracted Ion chromatograms (XIC) of all transition ions were integrated using a Skyline document (Skyline version 4.1.0.11796 https://brendanx-uw1.gs.washington.edu/labkey/project/home/software/Skyline/begin.view^44^. Relative peptide abundance was reported as a ratio of light to heavy peak area.

### Statistics

Statistical analysis was performed using GraphPad Prism 8 (GraphPad Software Inc.). Differences in ORF1p immunoreactivity or composite scores among morphologically normal FTE, STIC, and invasive HGSOC were examined using the Kruskal–Wallis test, followed by Dunn’s test for multiple comparisons of groups. Statistical analysis of the iMRM quantification results was performed using MSstats v3.7.3 at the peptide level on the log2 intensities of the endogenous peptides for each transition after normalization to the corresponding stable isotope labeled standard peptide. Data were exported from Skyline and custom formatted to allow MSstats to analyze the iMRM data separately by peptide for each protein. Peak areas from all samples were included regardless of whether endogenous levels of peptide were detected by manual inspection of the XICs.

### Ethics approval and consent to participate

The study was conducted in accordance with the Declaration of Helsinki. This study was approved by the Institutional Review Board at the Brigham and Women’s Hospital (BWH), Massachusetts General Hospital (MGH), Boston, MA, and the University of Pennsylvania (UPenn). All protocols for blood collection were approved by the Massachusetts General Hospital Institutional Review Board, and all subjects gave written informed consent.

## Results

### LINE1 ORF1p is detectable in conditioned media of HGSOC and is complementary to other known biomarkers

**Figure 1:**
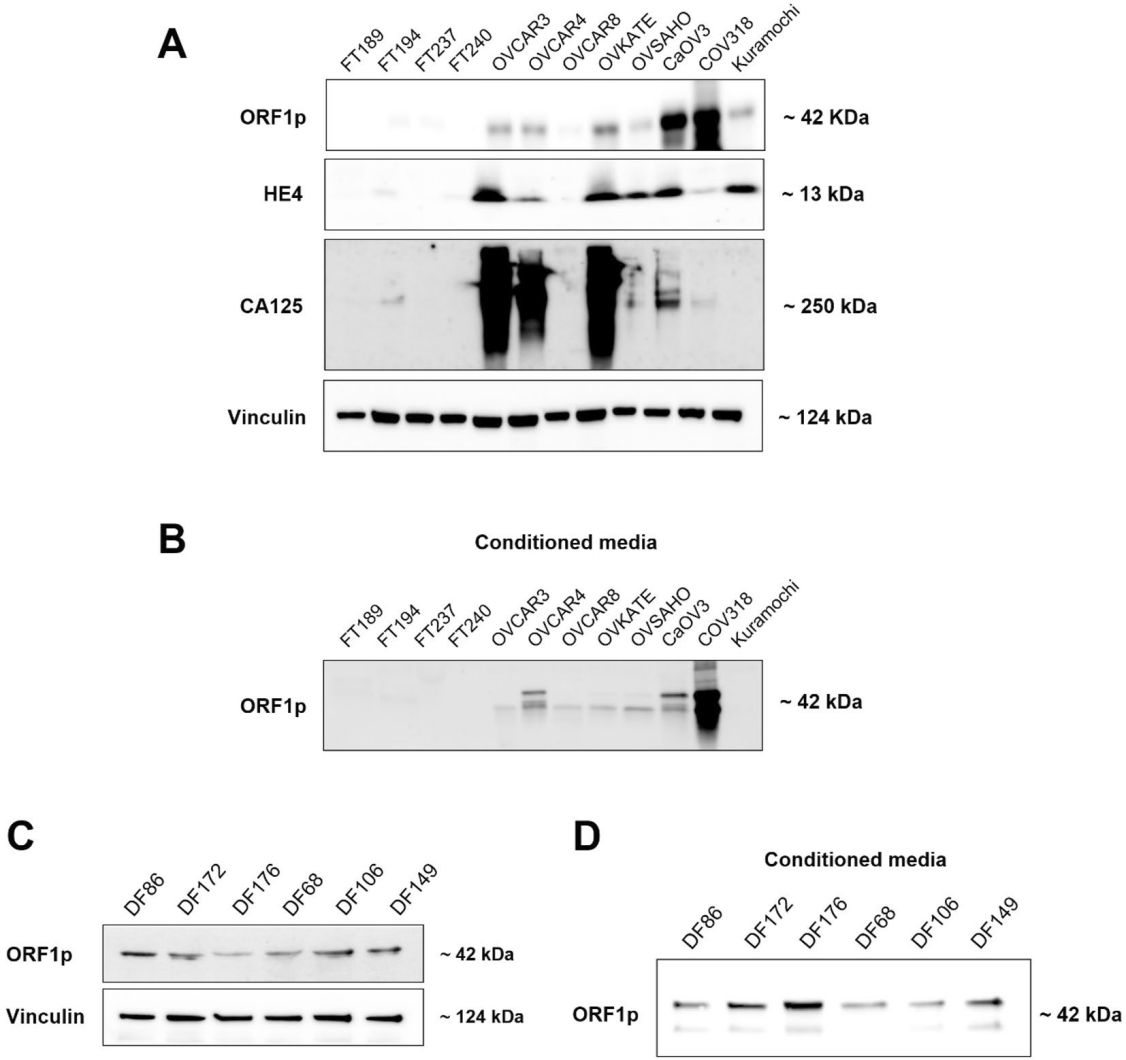
LINE1 ORF1p is detectable in conditioned media of HGSOC and is complementary to other known biomarkers. (**A**) Whole cell lysates. ORF1p, HE4, and CA125 protein expression (WB) in FT and HGSOC cell lines. Analyzed markers show differential expression between FT and HGSOC cells. Vinculin was used as internal control. (**B**) ORF1p protein expression (WB) in conditioned serum-free media from FT and HGSOC. (**C**) Whole cell lysate. ORF1p protein expression (WB) in six primary HGSOC cells derived from ascites fluid (DF cells). Vinculin was used as internal control. (**D**) ORF1p protein expression (WB) in primary HGSOC cells in conditioned serum-free media.

Restricted expression of LINE-1 ORF1p to tumorigenic tissue has been shown in a variety of cancers, including ovarian. Since cancer-specific proteins may serve as potential biomarkers, we evaluated ORF1p expression in the context of two FDA-approved ovarian cancer biomarkers, HE4 and CA-125. As expected, only cancer cell lines expressed ORF1p, HE4, and CA-125. Particularly, we observed that each marker exhibited a unique pattern of expression across the different cell line lysates (Fig. 1A). For instance, although ORF1p was expressed in COV318 cells, these cells did not express HE4 or CA-125. Similarly, OVSAHO cells uniquely expressed HE4 but negligible ORF1p or CA-125 (Fig. 1A). Since ORF1p expression is observed across a wide range of ovarian cancer samples and is independent of HE4 and CA-125, we postulate that ORF1p may serve as a useful analyte in a multi-modal, multi-biomarker screening tool for ovarian cancer.

To further test the potential of ORF1p as biomarker, we assessed whether ovarian cancer cells can release ORF1p into the media. For this purpose, we analyzed the conditioned media of cells in culture. Predictably, none of the FT cell lines released ORF1p. On the contrary, ovarian cancer cell lines release readily detectable ORF1p, except for Kuramochi and OVSAHO (Fig. 1B), which also displayed lower expression of ORF1p in whole cell lysates (Fig. 1A). To assess whether the ORF1p expression and release seen in HGSOC cell lines were artifacts of cell culture, we also examined ORF1p in primary tumor cells derived from ascitic fluid of patients with advanced HGSOC (DF cell lines^45^). Indeed, we observed that all six primary DF cell lines had detectable ORF1p expression (Fig. 1C) and release ORF1p into conditioned media (Fig. 1D). Taken together, ORF1p expression is limited to cancer cells, and its liberation into cell media makes ORF1p a good candidate for investigating its potential as biomarker.

### LINE-1 ORF1p is detectable in the plasma of ovarian cancer patients

The release of ORF1p by HGSOC cells led us to ask whether ORF1p could be detected in biological fluids of patients with the disease. To address this possibility, we developed immuno-multiple reaction monitoring followed by mass spectrometry (iMRM-MS) assays to detect two peptides that uniquely identify ORF1p (see “Methods” Section).

First, to assess assay performance, we prepared response curves in a standard plasma background, using endogenous peptide signal present in the sample as a reference. Heavy-labeled synthetic peptides from LINE-1 ORF1p were spike into 10 μL of digested plasma ranging at levels from 5 amol/μL to 50 pmol/μL in triplicate. Using the Quasar program in Skyline for response curve analysis and assuming an endogenous level of 1 fmol/μL in the sample, the limit of detection was 80 amol/μL for peptide LIGVPESDVENGTK, and 280 amol/μL for peptide LSFISEGEIK in plasma (Supplementary Fig. 7A and B).

**Figure 2:**
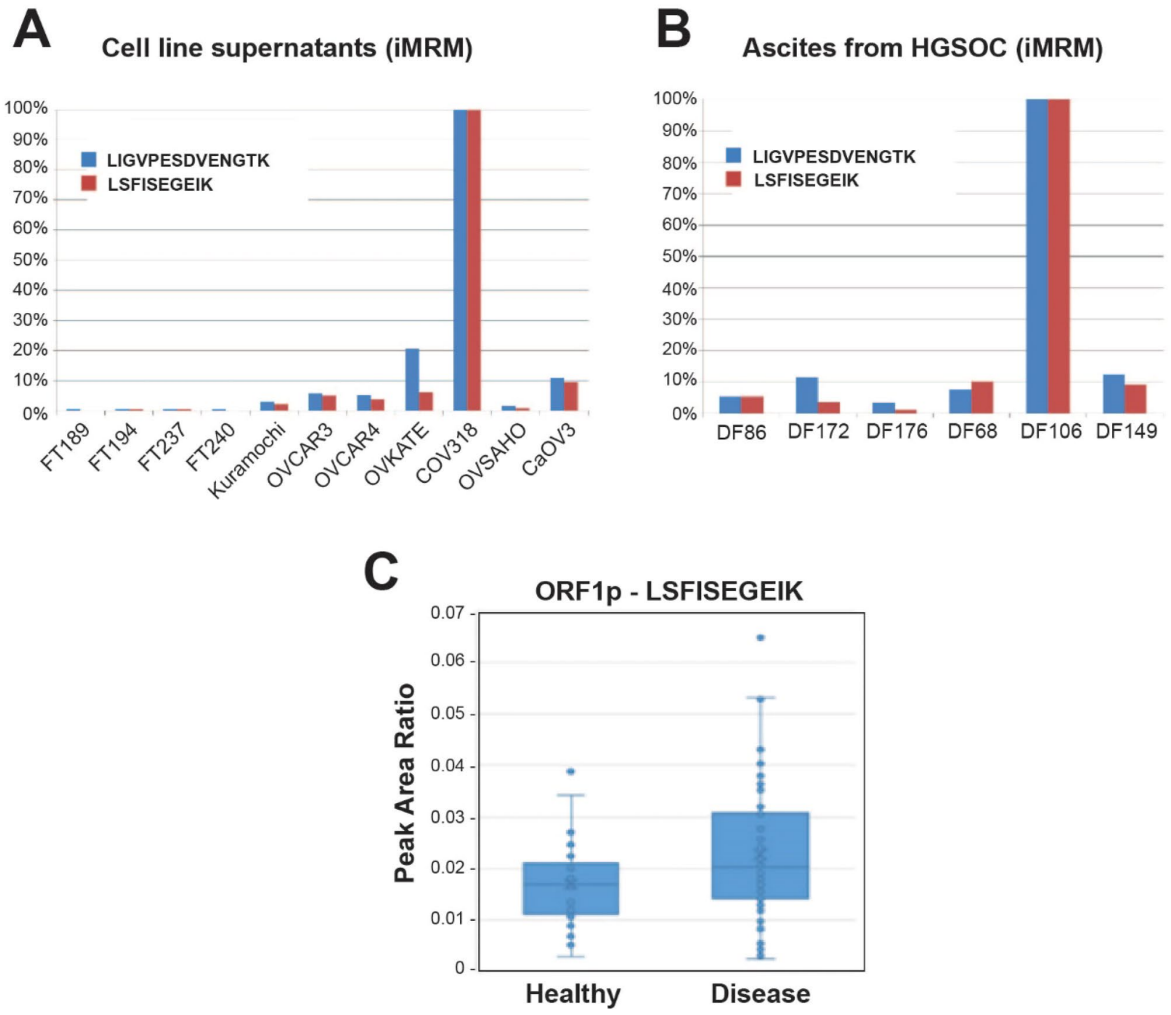
LINE-1 ORF1p is detected by iMRM-MS in conditioned media, ascites and plasma of HGSOC patients. (**A**) FT and HGSOC cell lines supernatants and (**B**) primary HGSOC cells from patients’ ascites were analyzed by iMRM-MS. Light to heavy peptide peak area ratio (PAR) for the single best transition was normalized to amount of protein in each sample. PAR for each sample was normalized to the sample with the highest value and reported as a percentage for each peptide. (**C**) Light to heavy peptide peak area ratio showing the relative detection and difference between healthy and disease samples of an independent cohort containing 72 cases of HGSOC and 37 healthy controls (N = 109 total patient plasma samples).

Next, to evaluate if iMRM-MS provides a similar readout of ORF1p relative abundance to that from Western blot analyses and could be applied to measure it in complex biological fluids, we assessed ORF1p expression by iMRM-MS in companion sets of conditioned media from cell lines and primary patient cultures. In conditioned media, iMRM-MS results were very similar to what we found in Western blots, with concentrations of ORF1p highest in COV318 cell supernatants and undetectable in supernatants from healthy FT cell cultures (Fig. 2A). Conditioned media from primary human DF cell lines were not analyzed by iMRM-MS; however, ORF1p levels in the patient ascitic fluid samples from which those cell lines were derived were measured and detected in all six cases (Fig. 2B). Given substantial expected differences in intraperitoneal tumor burden and total ascites volumes between patients, there was a surprising degree of concordance in the relative intensities of ORF1p signal between supernatants from primary HGSOC cultures and paired ascites samples as measured by Western blot and iMRM-MS, respectively (Figs. 1D, 2B). However, the signals for DF106 were markedly different, ORF1p was an order of magnitude higher in DF106 than other ascites samples as measured by iMRM-MS (Fig. 2B), while its level was at the lower end of the distribution when measured in primary cell line supernatants by Western blot (Fig. 1D). Nonetheless, the data suggest that ORF1p levels are detectable by iMRM-MS in biological fluids, though DF106 results emphasize that additional factors may influence the measured abundance of ORF1p in complex biological matrices.

We further used iMRM-MS to assess ORF1p across sample types, including the source of the signal (STIC or HGSOC cells), ascitic fluid, and the peripheral circulation, by evaluating companion sets of samples from cell line conditioned media (serving as a proxy for peri-tumoral concentration), and ascites fluid or plasma from patients with predominantly advanced stages of HGSOC (Supplementary Fig. 7C–E) (Methods and Supplementary Table 3). As expected, the amount of ORF1p peptide MS signal was inversely correlated to the concentration of the background matrix. Using antibody enrichment of peptides followed by targeted MS confidently identified at least one of the ORF1p peptides, LSFISEGEIK, in approximately 10% of the plasma samples, albeit with 30 times lower signal intensity than in conditioned cell media (2500 vs. 75,000 cps) (Supplementary Fig. 7E vs. 7C). While dilution of the signal from source to circulation was likely a central factor, the concentration of the ORF1p peptides remained low even after antibody enrichment, possibly due to signal suppression from non-specific or low-affinity binding peptides with much higher endogenous abundance.

Additional experiments were performed to determine whether the detected signal (2500 cps) derived from endogenous protein in plasma or represented a technical artifact due either to incomplete isotopic labeling of the heavy peptide^38^ or to passenger peptide in the antibody (peptides bound to the polyclonal antibody during the antibody generation and affinity purification process; see “Methods” Section)^35^. As shown in Supplementary Fig. 8A, the intensity of light signal observed in a sample of heavy peptide was very low, less than 300 cps, and for any detectable signal, the ratio of transitions did not match that of the heavy standard. The light peptide signal intensity remained low and unchanged with antibody enrichment in buffer and control plasma, and in all cases had a transition ratio inconsistent with that of the heavy peptide. Thus, there was no evidence to support an artifact due to exogenous peptides. Though in patient plasma the light:heavy peak area ratio of ORF1p was below what is generally considered a quantifiable ratio (0.007), it was distinctly greater than the signal in the control test samples (Supplementary Fig. 8B).

Finally, we performed iMRM-MS assays on a cohort of 109 patient samples comprising 72 cancer and 37 healthy patients, in singlicate on three separate days, using the statistical framework provided in MSstats (see “Methods” Section)^46^. As shown in Fig. 2C, there was a trend of higher concentrations of ORF1p in HGSOC samples compared to the controls, albeit the fold change (log_2_ = 0.035) was not statistically significant.

Altogether, these results demonstrate that ORF1p can be confidently detected in the bloodstream of patients with HGSOC. Nevertheless, further improvements in the limits of quantification of the assay and a larger test population would be required to provide more precise quantitative estimates of the performance of ORF1p as a plasma diagnostic biomarker.

### LINE-1 ORF1p expression is an early event in serous ovarian cancer tumorigenesis

**Figure 3:**
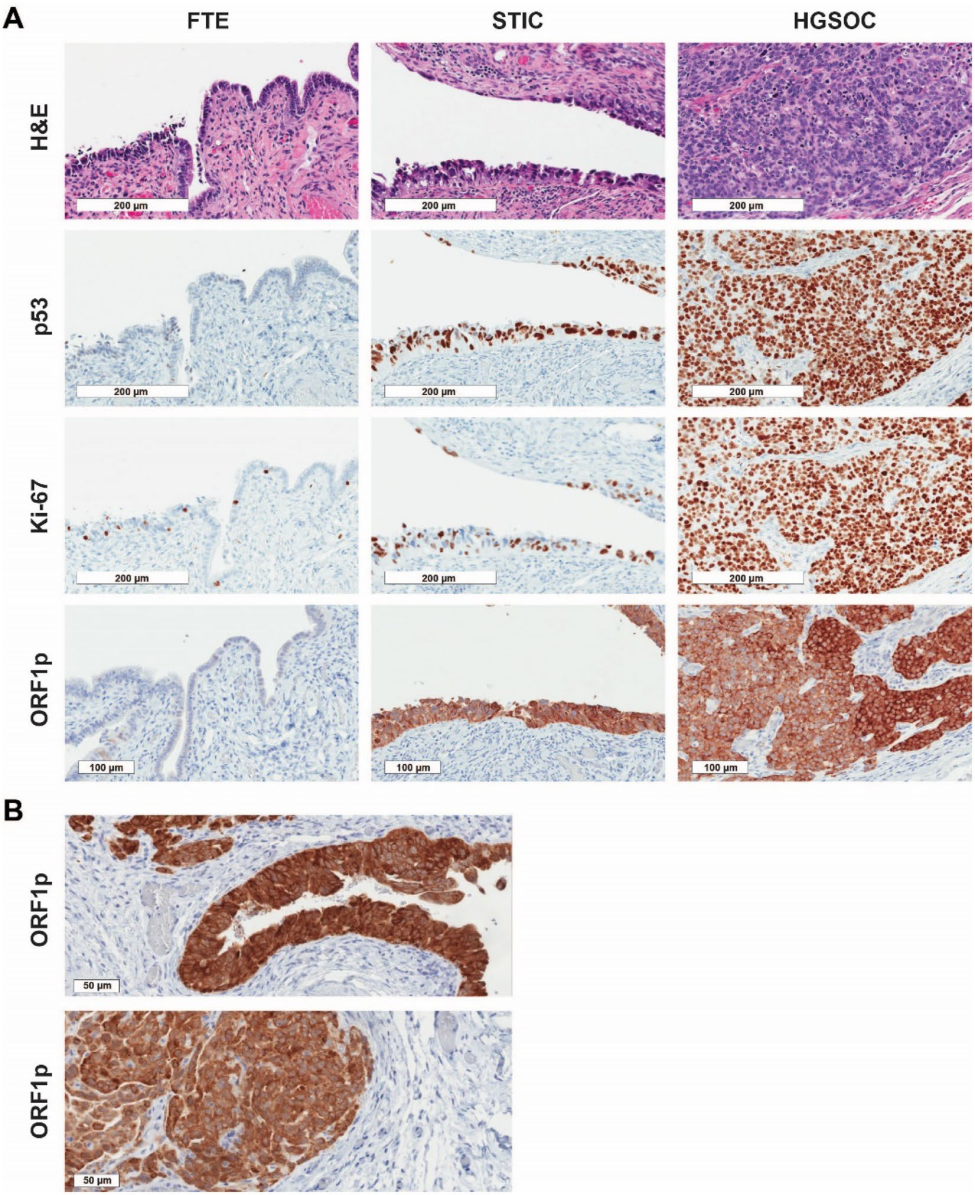
LINE-1 ORF1p expression is an early event in serous ovarian cancer tumorigenesis. (**A**) LINE-1 ORF1p expression (IHC) in tissue from morphologically benign fallopian tube epithelium (FTE), serous tubal intraepithelial carcinoma (STIC), and invasive high-grade serous ovarian carcinoma (HGSOC). Abundant ORF1p is expressed in STIC lesions and HGSOC while normal FTE is negative. p53 staining identifies the carcinoma cells and Ki-67 the proliferative cells in the STIC and invasive tumor. All micrographs (20X objective) were imaged from one representative case to align the location of the lesions. (**B**) Cellular distribution of ORF1p in HGSOC. Two representative cases are shown, which exhibit a cytoplasmic and membranous staining pattern (40X objective).

The observation that ORF1p is expressed by HGSOC cells and detectable in biological fluids, prompted us to investigate whether ORF1p expression is an early or late event in HGSOC tumorigenesis. We utilized immunohistochemistry (IHC) to assess ORF1p expression in tissue specimens from 30 patients diagnosed with HGSOC and 12 healthy controls. p53 and Ki-67 stains were performed to identify carcinoma cells and proliferative cells, respectively (Fig. 3A). In the HGSOC cases, one or more STICs were identified in 18 specimens, while adjacent, morphologically normal FTE was present in 25 cases. Normal FT epithelium was negative for ORF1p expression, while HGSOC was diffusely positive in almost all cases (Fig. 3A, Table 1). The lack of expression in FT epithelium was observed in cases with and without STIC or HGSOC, suggesting that normal benign FT epithelium is negative for ORF1p. Expression in HGSOC was generally robust and diffuse (Fig. 3A,B) with a cytoplasmic or pan-cellular distribution (Fig. 3B). Interestingly, when we dichotomized the IHC scores into negative (0 and 1) and positive (2 and 3) groups, expression in STIC lesions was robust with 14/18 cases showing positivity (Fig. 3A, Table 1).

**Table 1:** ORF1p expression in serous tubal intraepithelial carcinoma and HGSOC.

	ORF1p stain intensity			
	Negative		Positive	
	0 (%)	1 (%)	2 (%)	3 (%)
Normal FTE (n = 12)	12/12 (100)	0/12 (0)	0/12 (0)	0/12 (0)
Adjacent FTE (n = 25)	23/25 (92)	2/25 (8)	0/25 (0)	0/25 (0)
STIC (n = 18)	2/18 (11)	2/18 (11)	5/18 (28)	9/18 (50)
Invasive HGSOC (n = 30)	0/30 (0)	1/30 (3)	5/30 (17)	24/30 (80)

*FTE* Fallopian tube epithelium, *STIC* serous tubal intraepithelial carcinoma, *HGSOC* high-grade serous ovarian carcinoma.

**Figure 4:**
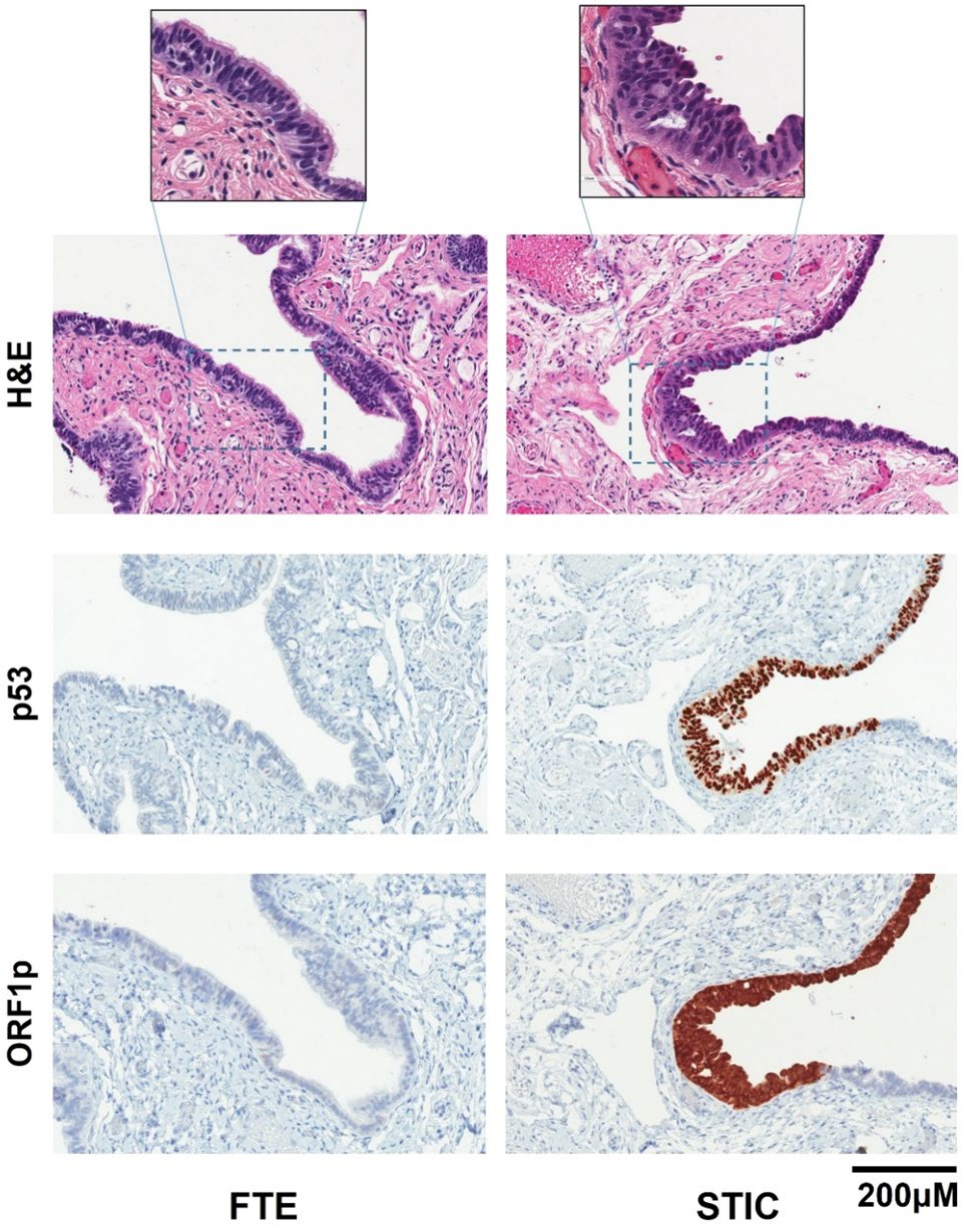
LINE-1 ORF1p is expressed in incidental STIC lesions. Representative images of hematoxylin and eosin (H&E) staining, p53, and LINE-1 ORF1p expression in fallopian tube epithelium (FTE) and serous tubal intraepithelial carcinoma (STIC) lesions (IHC). Incidental STIC lesions display robust cytoplasmic and pan-cellular ORF1p expression (20X). There is no ORF1p expression in the adjacent benign epithelium. Intense nuclear p53 staining is characteristic of the STIC lesion and is negative in normal tissue. Inset micrographs (40X) highlight the normal ciliated cells in benign FTE and the malignant cells in the STIC lesion.

Recent genomic studies of FT precursors and HGSOC have shown that in cases of advanced HGSOC, the presence of what appears to be STIC in the FT may actually be metastatic disease masquerading as a precursor^47–50^. To address this possibility, we stained six cases of incidental STIC lesions from risk-reducing surgeries for ORF1p expression. In these cases, there is no invasive disease. ORF1p expression was detected in four of the six cases. As in the cases with HGSOC, ORF1p expression was robust in the malignant STIC cells and not in the adjacent normal FT epithelium (Fig. 4). These results confirm that ORF1p expression occurs in FT precursors to HGSOC.

### Loss of DNA Methylation in fallopian tube cells leads to LINE-1 ORF1p expression and detection in conditioned media

**Figure 5:**
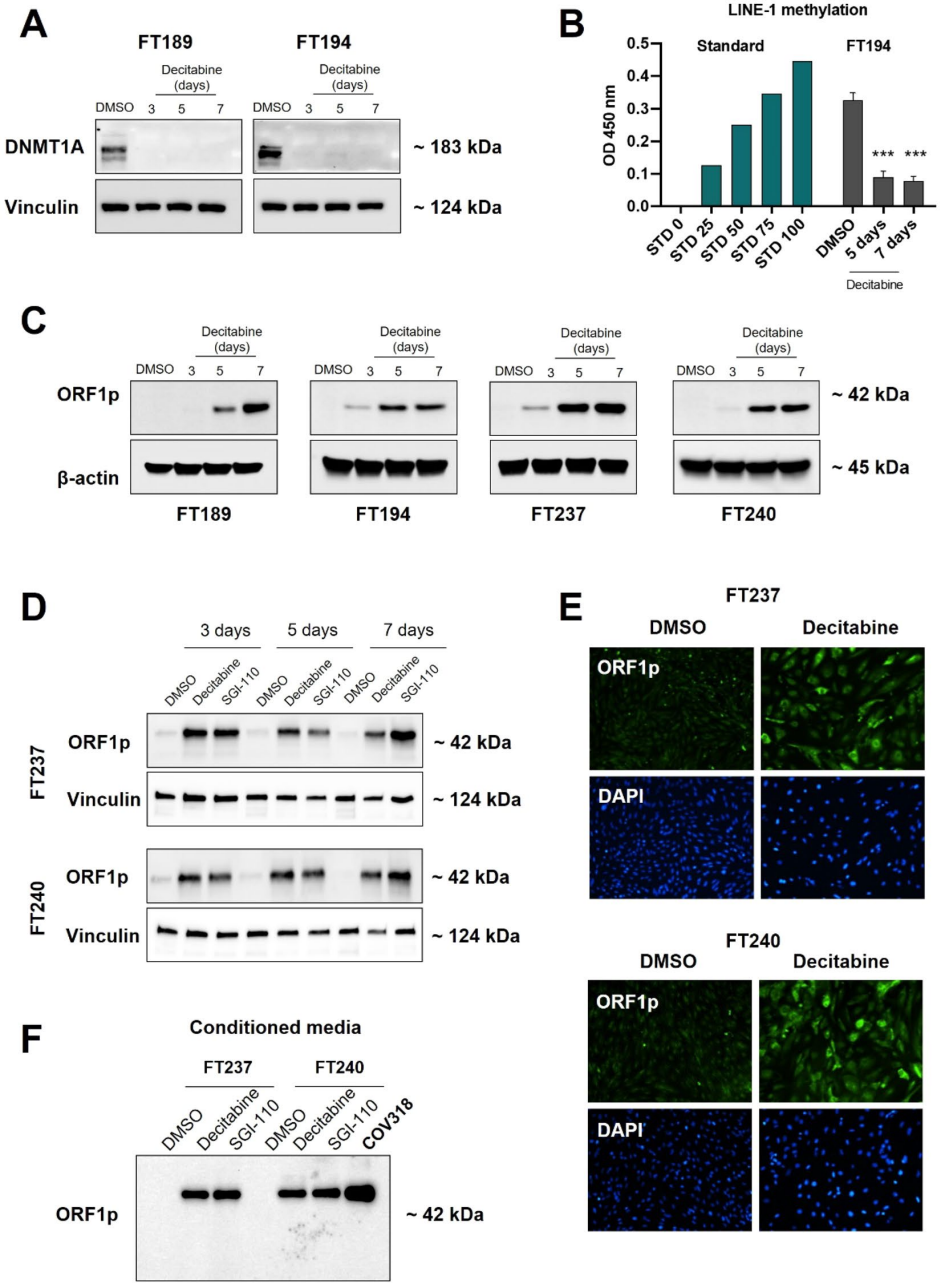
Loss of DNA Methylation in fallopian tube cells leads to LINE-1 ORF1p expression and detection in conditioned media. FT cell lines were treated with DNMT inhibitors Decitabine or SGI-110 (5 µM) for 3, 5 or 7 days. DMSO was used as negative control. (**A**) DNMT1A protein expression (WB) and (**B**) LINE-1 methylation levels after Decitabine treatment. 5-methylcytosine levels of LINE-1 in genomic DNA were quantified and the optical density (OD) was measured at 450 nm. A significant decrease in DNMT1A levels and LINE-1 methylation (N = 3. ***p < 0.0002) was observed. (**C**) ORF1p protein expression (WB) in FT cells after Decitabine treatment. None of the lines expressed ORF1p prior to treatment but ORF1p was abundantly expressed in all the lines after 5 days of treatment. β-actin serves as the loading control. (**D**) Comparison of LINE-1 ORF1p expression in FT cells after Decitabine or SGI-110 treatment by WB. Both compounds are equally capable of inducing ORF1p expression as early as 3 days. (**E**) FT cells treated with Decitabine or SGI-110 were examined by immunofluorescence for the presence of ORF1p (10X objective). (**F**) ORF1p protein expression (WB) in conditioned media of FT cells after treatment with demethylating agents. COV318 was used as a positive control for ORF1p release.

It is well documented that in normal somatic cells, DNA methylation and related mechanisms inhibit LINE-1 retrotransposon expression. In neoplastic cells, however, DNA is commonly hypomethylated, leading to the increased LINE-1 expression observed in a range of cancers, including ovarian^26,51,52^. To assess whether DNA demethylation could induce LINE-1 expression and release in non-tumorigenic FT cells, we treated four different FT cell lines with the DNA methyltransferase inhibitor (DNMTi) decitabine (5 μM) for 3, 5, or 7 days. As expected, decitabine treatment led to depletion of DNMT1A (Fig. 5A) and to a decrease in LINE-1 methylation in FT cell lines (Fig. 5B). Regarding ORF1p, all four FT lines showed a robust ORF1p expression after the treatment, while DMSO alone had no effect (Fig. 5C). Moreover, we tested a second generation DNMTi, SGI-110 (Guadecitabine), which was rationally designed to be resistant to degradation by cytidine deaminase and to prolong the exposure of cells to the active metabolite, decitabine, ensuring greater uptake into the DNA of rapidly dividing cells^53,54^. Consistent with our previous results, treatment with 5 μM SGI-110 lead to strong expression of ORF1p as early as 3 days after treatment (Fig. 5D). Prolonged treatment for 5 or 7 days led to further enhancement of ORF1p expression, while DMSO had no effect (Fig. 5D). Immunofluorescent microscopy confirmed the expression of ORF1p in decitabine treated versus untreated FT cells. Consistent with the intracellular localization of ORF1p in HGSOCs, ORF1p was found predominantly in the cytoplasm of DNMTi-treated FT cells (Fig. 5E).

Lastly, we asked whether treatment of FT cells with decitabine or SGI-110 promotes the release of ORF1p. For this purpose, we treated FT cells with DMSO, Decitabine, or SGI-110 for 5 days and analyzed their conditioned media. Consistent with our cell line data, treatment of FT cells with DNMTis led to ORF1p secretion (Fig. 5F). Together, these data indicate that DNA methylation is a necessary mechanism to restrain LINE-1 expression and ORF1p release in FT cells.

## Discussion

The human genome is replete with interspersed repetitive elements that reflect the activity of transposable elements. LINE-1 is such a sequence; a mobile genetic element active in humans that is self-propagating and protein-coding^19,51,55^. LINE-1 sequences are not only an important source of heritable structural variation, but they can also lead to acquired insertions in cancer genomes^19^. Here, we report on the expression of ORF1p, one of the protein-coding products of LINE-1, in high-grade serous ovarian cancer. We use different approaches to make three main observations. First, we show that ORF1p is expressed and released by tumor cells and primary ascites-derived HGSOC cell lines and that it can be confidently detected in biological fluids including ascites and plasma from patients with ovarian cancer using iMRM-MS. Second, we show that ORF1p expression is an early event in HGSOC development, as it is expressed in FT precursor and, in particular, in incidental STIC lesions. Lastly, we show that DNA demethylation can activate ORF1p expression in immortalized FT epithelial cells, consistent with DNA methylation acting as a necessary mechanism to suppress LINE-1 expression in these benign cells.

A wide range of cancers has been reported to express LINE-1 ORF1p, including renal, esophageal, pancreatic, lung, prostate, breast, and ovarian carcinomas^26,27,29,56,57^. Here, we report that ovarian cancer cells not only express but also release ORF1p, being highly relevant from the perspective of clinical biomarker development. In this context, we developed immunoMRM-MS assays to detect proteotypic ORF1p peptides found in the tissue interstitial fluid of HGSOC tissue samples (Gillette et al., manuscript in preparation). We used iMRM-MS assays since this approach has several key features: (1) the measurement of proteins using a format that supports high-level multiplexing of biomarker candidates with efficient sample processing; (2) the use of affinity reagents (anti-peptide antibodies) increases the relative abundance of the proteotypic peptide targets, enhancing analytical sensitivity of their detection in complex biofluids commonly collected and measured in the clinic (e.g., plasma); and (3) the use of mass spectrometry in place of a second antibody provides sequence specificity, ensuring that the measurement derives from the intended analyte^34^.

iMRM-MS assays largely recapitulated Western blot findings in supernatants from both commercial and primary cell lines. Notably, iMRM-MS demonstrated a decremental signal gradient as distance from the signal source increased from peri-tumoral samples to ascites to plasma, much as has been described for CA-125 levels moving from ovarian cyst fluid to ascites and plasma in women with HGSOC^58^. The general correspondence between relative ORF1p levels in primary cell line supernatants and their matched ascites samples suggests that differential levels might serve as a diagnostic biomarker in accessible body fluids, but only systematic testing in large numbers of HGSOC patient plasma samples and suitable controls can adequately test this hypothesis.

While iMRM-MS results for both configured peptide assays were highly correlated and both worked well in supernatants, only the LSFISEGEIK assay detected endogenous ORF1p in patient plasma samples. When additional samples were analyzed by iMRM-MS from plasma that was immunoaffinity-depleted of the highest-abundance plasma proteins (data not shown) the relative number of samples where ORF1p was detected increased threefold, to ~ 30%. Despite these advances, and though detection in plasma can be confidently claimed, the low absolute intensity of the ORF1p signal and corresponding low light:heavy peak area ratios preclude accurate quantification in these samples. Furthermore, as ORF1p signals are only marginally above noise in samples in which they are detected, no strong claims can be made about samples in which ORF1p was not detected. These limitations notwithstanding, our results certainly contribute to maturing the potential implementation of ORF1p as an ovarian cancer biomarker and highlight the need for still more sensitive assays for the study of plasma biomarkers.

Quantification of LINE-1 in biological fluids has been a matter of study in a number of prior publications, emphasizing its potential as cancer biomarker. Regarding LINE-1 DNA, the assessment of LINE-1 by qPCR in circulating DNA in breast cancer patients’ sera has been shown valuable for detecting early-stage breast cancer^59^. In terms of LINE-1 DNA methylation, studies showed using cell-free DNA (cfDNA), that melanoma serum samples had significantly higher unmethylated LINE-1 levels than healthy donor serum^60^. Similarly, LINE-1 hypomethylation of plasma cfDNA was proposed as a disease progression biomarker for colorectal cancer^61^. Recently, a study used digital ELISA and droplet microfluidics (ddELISA) to detect ORF1p in serum samples from breast cancer patients. This approach was more sensitive than the current gold standard for ultrasensitive protein detection^62^. Together, our and previous findings encourage the study and development of LINE-1 and ORF1p as a marker in ovarian cancer.

The stage of neoplastic transformation at which LINE-1 elements are activated is not clearly understood. Recent reports^30,31^ suggest that the normal restraints on LINE-1 expression are lost early in tumor development. Our results are consistent with those findings, as we observed that while ORF1p is negative in benign FT epithelium, is present in early non-invasive human HGSOC precursor lesions (STICs) and maintained throughout HGSOC progression. Importantly, the evaluation of ORF1p expression in incidental STIC lesions uniquely demonstrates that its expression is a manifestation of early rather than metastatic disease.

DNA methylation of LINE-1 elements has been postulated as a major mechanism of suppression in normal adult tissues. In this regard, we tested whether DNA demethylating agents could induce ORF1p expression and release in FT cell lines. During replication, decitabine is incorporated into DNA where it can covalently trap DNMT enzymes, creating DNA–protein adducts^63^, and subsequently DNMTs degradation^64^. Our data showed that the treatment of FT cells with DNMT inhibitors, Decitabine and SGI-110, was sufficient to trigger ORF1p expression and release into conditioned media.

Although p53 has been postulated as a critical suppressor of LINE-1 expression and activity in somatic human cells^57,65^, our data suggest that p53 deficiency alone does not lead to LINE-1 derepression and ORF1p expression. FT cell lines used in this study were immortalized by disrupting the p53 pathway^41,42^, FT189 and FT194 were immortalized using human telomerase reverse transcriptase (hTERT) and SV40 T-antigen, while FT237 and FT240 were immortalized without viral oncoproteins, and none of these lines express ORF1p. Moreover, we and others have not detected ORF1p expression in the earliest fallopian tube lesions that harbor *TP53* mutations, known as p53 signature^30,31^, suggesting the involvement of additional regulatory mechanisms.

In summary, ORF1p appears to be uniquely expressed by ovarian cancer cells compared to fallopian tube cells. The presence of ORF1p in incidental STIC lesions indicates that its expression is an early event in tumorigenesis. The apparent binary expression of ORF1p is regulated by DNA methylation which is lost during neoplastic transformation and contributes to the release of ORF1p into biological fluids. Although more sensitive assays are needed, the confident detection of ORF1p in biological fluids from patient plasma supports it further development as a candidate biomarker for ovarian cancer.

## Supplementary Information

Supplementary Information.

## Data Availability

Further information and requests for resources and reagents should be directed to the Lead Contact, Dr. Ronny Drapkin at rdrapkin@pennmedicine.upenn.edu. This study generated new LINE-1 ORF1p peptide antibodies.
