# Supplementary material for: LINE-1 ORF1p as a candidate biomarker in high grade serous ovarian carcinoma
Source: Sci Rep. 2023 Jan 27;13:1537. doi: 10.1038/s41598-023-28840-5 (PMC9883229; doi:10.1038/s41598-023-28840-5)
Supplement: Supplementary file 1 — Supplementary Information. [file 41598_2023_28840_MOESM1_ESM.pdf]

LINE-1 ORF1p as a candidate biomarker in high grade serous ovarian carcinoma

Sho Sato*, Michael Gillette*, Pamela R. de Santiago, Eric Kuhn, Michael Burgess, Kristen Doucette, Yi Feng, Carlos Mendez-Dorantes, Paul J. Ippoliti, Sara Hobday, Marilyn A. Mitchell, Kai Doberstein, Stefan M. Gysler, Michelle S. Hirsch, Lauren Schwartz, Michael J. Birrer, Steven J. Skates, Kathleen H. Burns, Steven A. Carr, Ronny Drapkin

Supplementary Information

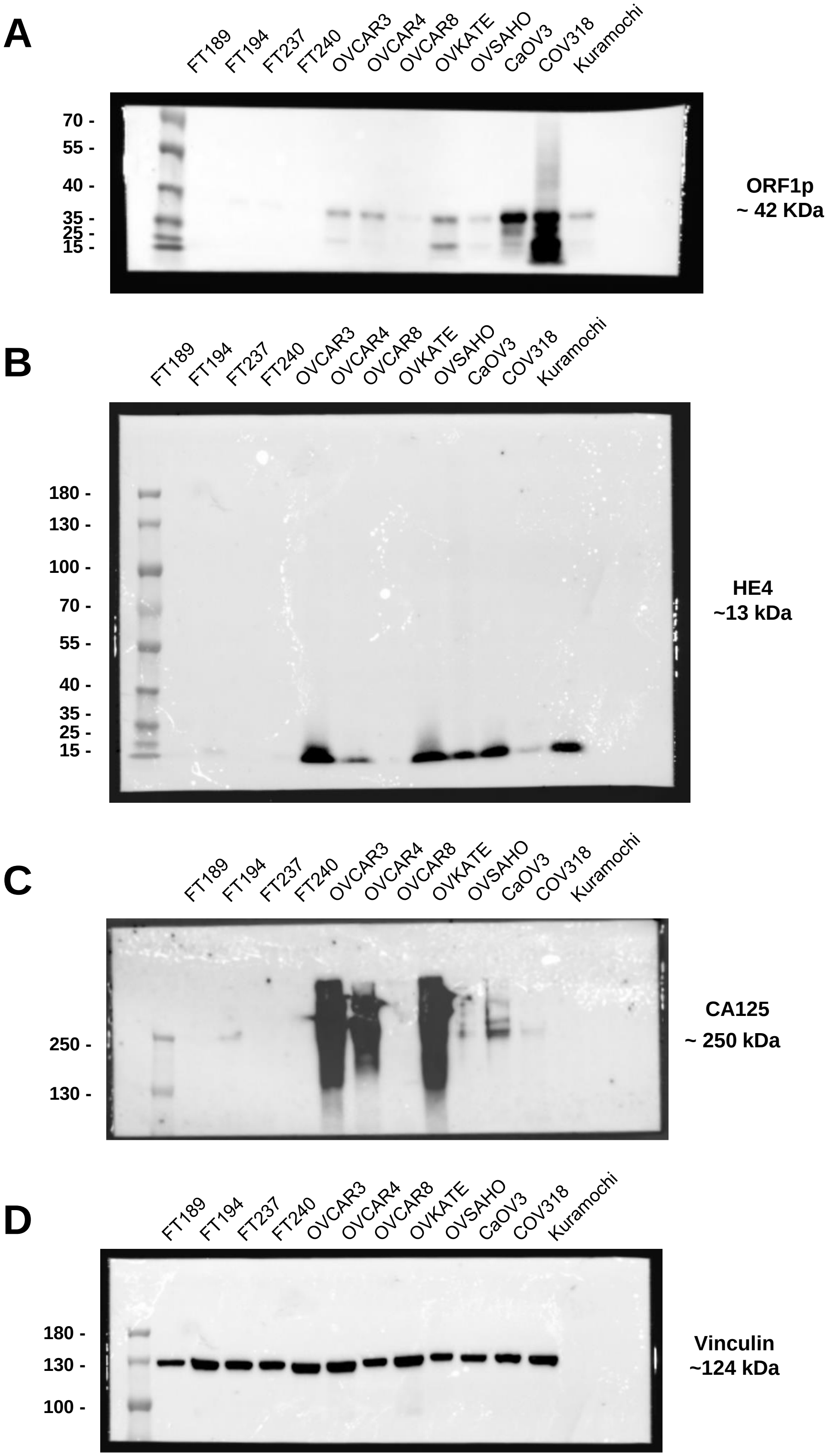

Supplementary Figure 1. Extended data from Figure 1A. (A) Uncropped ORF1p membrane, **(B)** uncropped HE4 membrane, **(C)** uncropped CA125 membrane, and **(D)** uncropped Vinculin membrane. Chemiluminescent and Color images were merged to show molecular weight standard (Thermo Scientific, Cat# 26616).

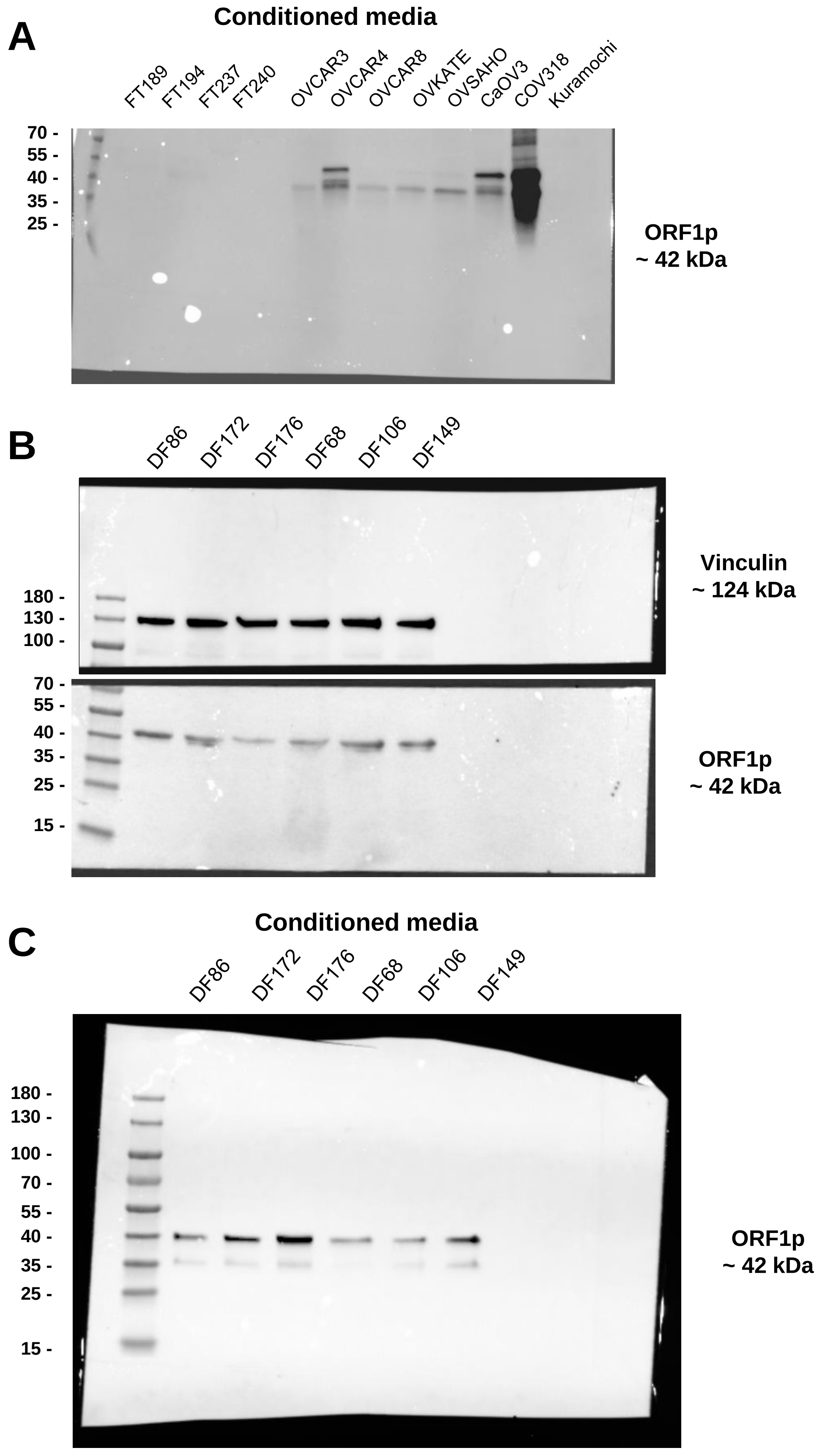

Supplementary Figure 2. Extended data from Figure 1B, C and D. (A) Uncropped ORF1p membrane from Fig. 1B. **(B)** Uncropped Vinculin and ORF1p membranes from Fig. 1C. **(C)** Uncropped ORF1p membrane from Fig. 1D. Chemiluminescent and Color images were merged to show molecular weight standard (Thermo Scientific, Cat# 26616).

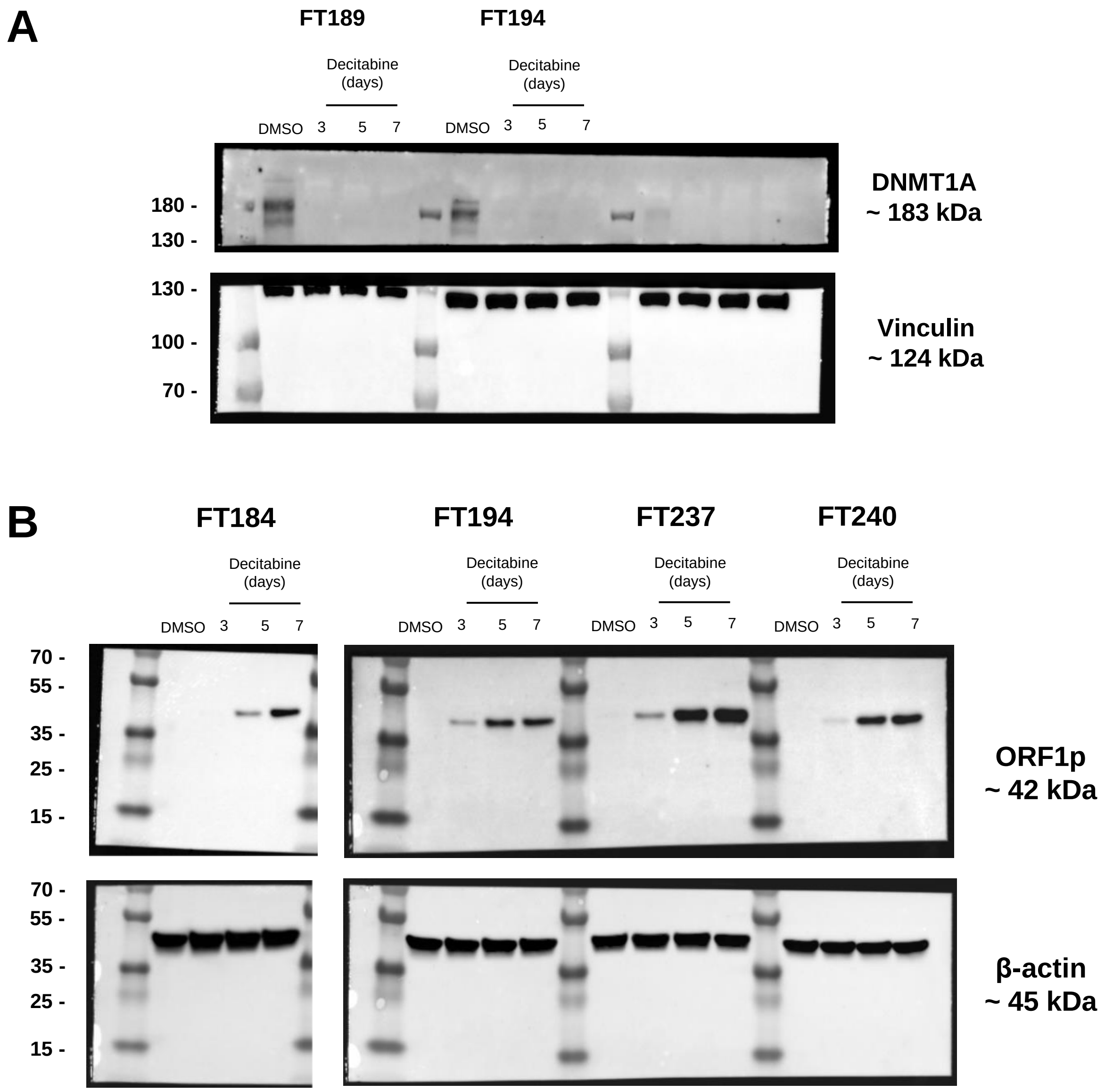

Supplementary Figure 3. Extended data from Figure 5A and C. (A) Uncropped DNMT1A and Vinculin membranes from Fig. 5A. **(B)** Uncropped ORF1p and β-actin membranes from Fig. 5C. Membranes were stripped and then re-probe. Chemiluminescent and Color images were merged to show molecular weight standard (Thermo Scientific, Cat# 26616).

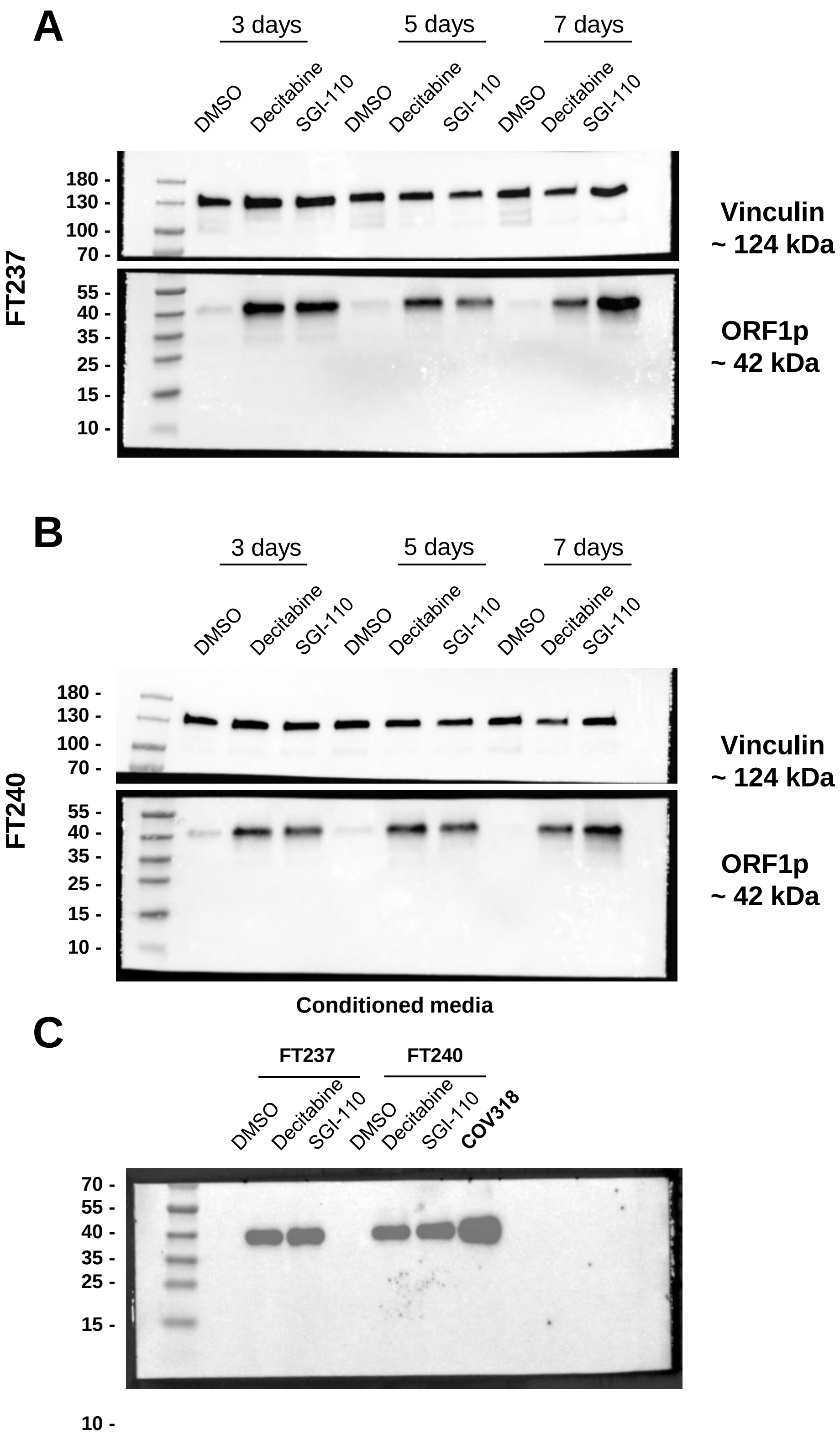

Supplementary Figure 4. Extended data from Figure 5D and F. Uncropped ORF1p and Vinculin membranes of **(A)** FT237 and **(B)** FT240 from Fig. 5D. **(C)** Uncropped ORF1p membrane from Fig. 5F. Chemiluminescent and Color images were merged to show molecular weight standard (Thermo Scientific, Cat# 26616).

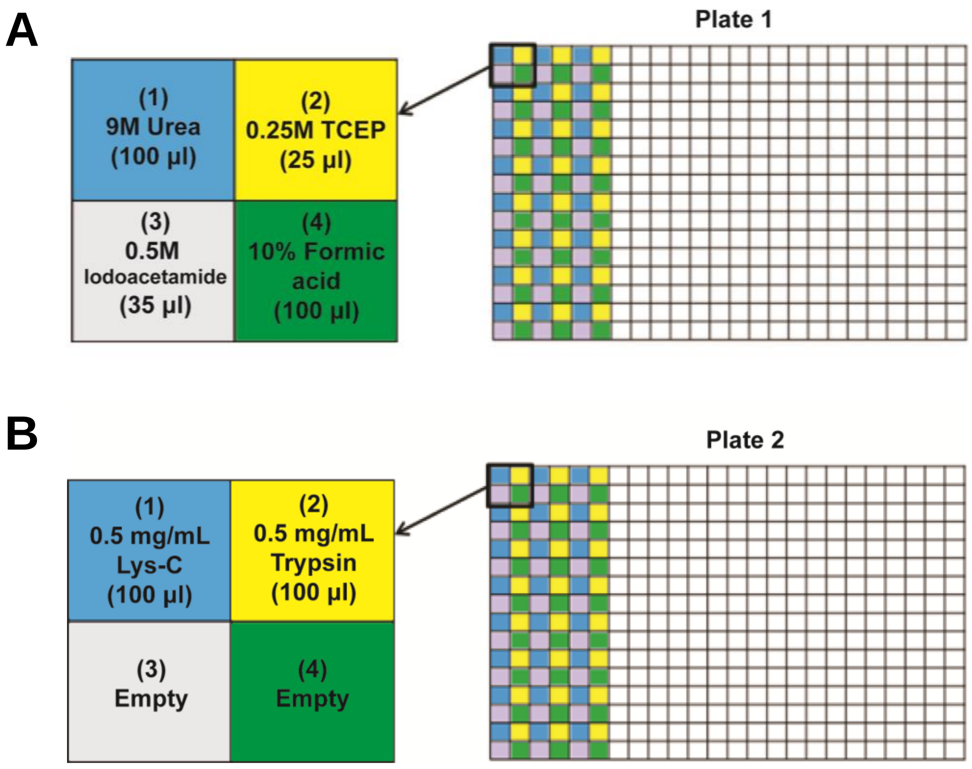

Supplementary Figure 5. **(A)** Example diagram of the locations for the digestion reagents, 9 M Urea, 0.25 M TCEP, 0.5 M IAA and 10% formic acid, as added into the first 384 well plate designated "plate 1". Only wells that corresponded to sample wells contained reagents. Once filled, plate 1, Digestion Reagents, was placed into position 7 on the Bravo LT (see Supplementary Fig. 6). **(B)** Example diagram of the locations for the enzymes used to digest plasma, lys-C followed by trypsin, as added into the second 384 well plate designated "plate 2". Only wells that corresponded to sample wells contained reagents. Once filled, plate 2, Enzymes, was placed into position 6 on the Bravo LT (see Supplementary Fig. 6).

(1) 250 µl Transfer tips	(2) 250 µl Transfer tips	(3) 250 µl Transfer tips
(4) Temperature Controlled shaker	(5) Sample plate	(6) Enzymes (384-well plate)
(7) Digestion reagents (384-well plate)	(8) 250 µl liquid Transfer tips	(9) 0.2M Tris-HCl (96-well plate)

Supplementary Figure 6. Bravo LT diagram used for plasma digestion. Bravo deck positions as numbered contained the either the transfer tips, reagent plates or solvent plates for digestion. Plasma was added to the 96 deep well sample plate and placed onto position 5. After each reagent addition step, the robot moved the sample plate to position 4 where the plasma samples were mixed and incubated as described in Methods.

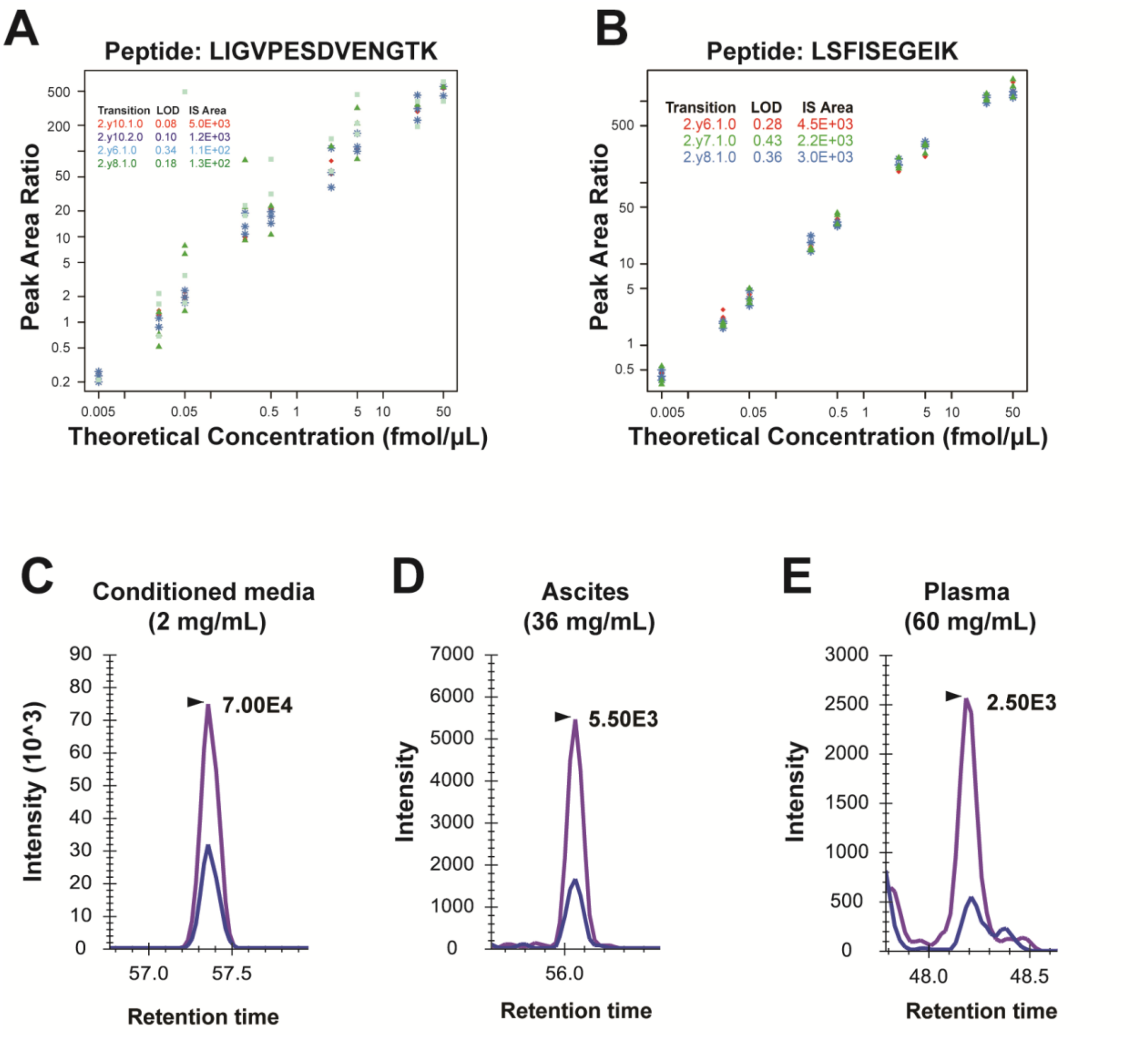

Supplementary Figure 7. Response curves across a range of heavy peptide spike concentrations for the best transitions for ORF1p peptide **(A)** LIGVPESDVENGTK and **(B)** LSFISEGEIK are used to determine limits of detection (LODs) assuming an endogenous concentration of 1 fmol/μL plasma. LODs are calculated using the measurement level and variation observed in the blank and low concentration samples. **(C)** Extracted ion chromatograms (XICs) for two (purple and blue lines) light (endogenous) transitions for ORF1p peptide LSFISEFEIK for representative samples of conditioned cancer cell line serum-free media (total protein concentration = 2 mg/mL), **(D)** patient ascites fluid (total protein concentration = 36 mg/mL), and **(E)** plasma (total protein concentration = 60 mg/mL). The y8 transition ion 561.80/922.48 was removed due to an interference in the plasma samples. While representative, samples were not isolated from the same patient.

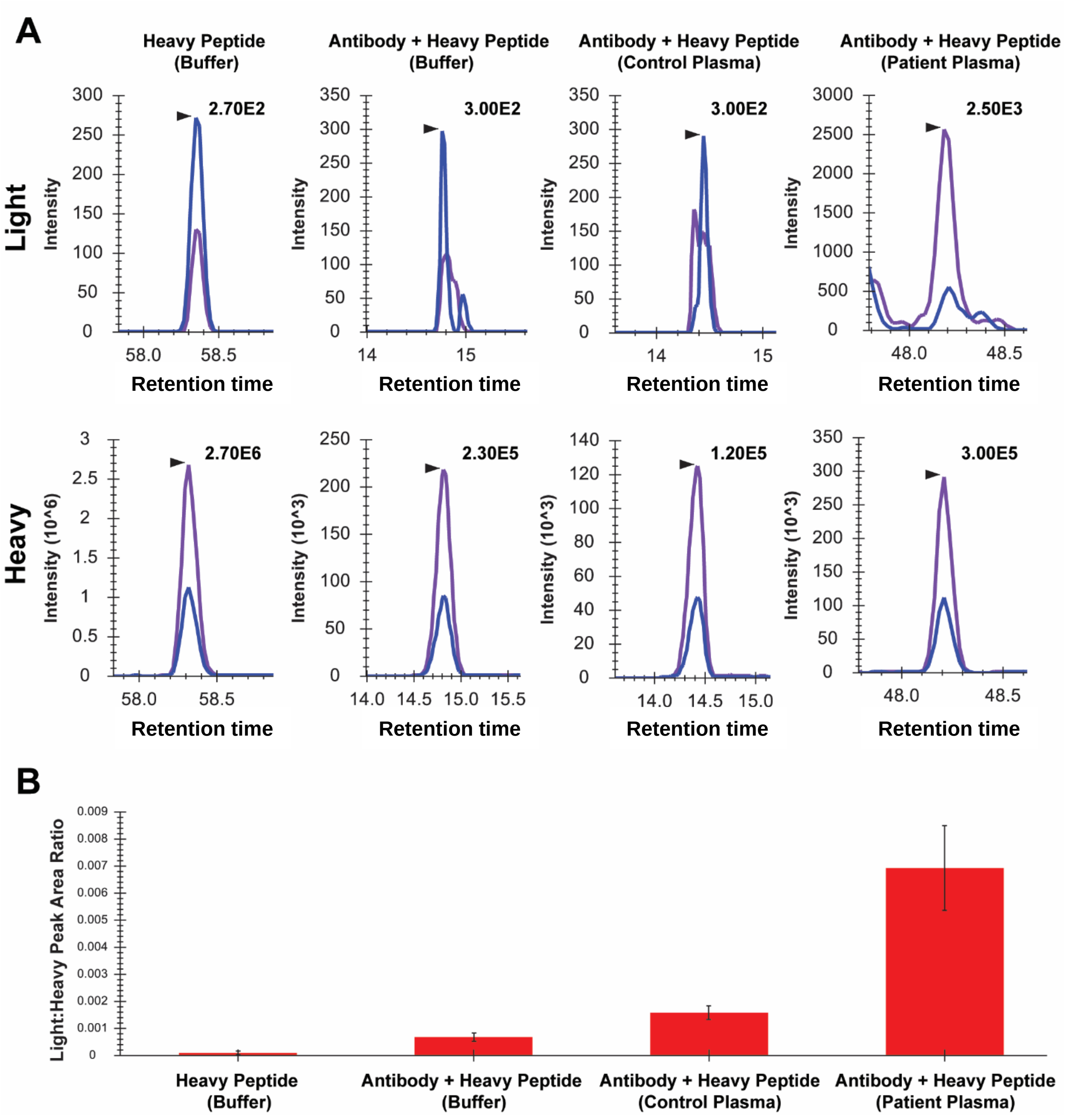

Supplementary Figure 8. (A) Extracted ion chromatograms (XICs) for two transitions for the light (endogenous) and heavy standard versions of ORF1p peptide LSFISEGEIK monitored in samples containing heavy peptide standard alone, heavy peptide standard immunoaffinity-enriched from buffer or control plasma, or heavy peptide standard spiked into patient plasma. Observed maximum transition intensities (peak heights) denoted by the arrowheads are listed in scientific notation. The y8 transition ion 561.80/922.48 was removed due to an interference in the plasma samples. **(B)** Light to heavy peak area ratio as calculated in Skyline for the corresponding samples. Error bars represent standard error.

Supplementary Table 1. Clinical characteristics of the 72 ovarian cancer patients studied

Clinical feature	Percentage
**Ovarian cancer classification**	
Papillary serous carcinoma	89%
Serous borderline	11%
**Stages**	
Stage I	10%
Stage II	4%
Stage III	60%
Stage IV	25%
**Grade**	
Grade I	3%
Grade II	12%
Grade III	75%
N/A	8%

Supplementary Table 2. List of cell lines used in these studies

Cell lines	Cell line origin	Media
Fallopian tube cell lines	Drapkin lab	DMEM/F12 + 2% USG
DF-cell lines	Drapkin lab	RPMI 1640 + 10% FBS
KURAMOCHI	Japanese Collection of Research Bioresources Cell Bank	RPMI 1640 + 10% FBS
OVCAR3	ATCC	RPMI 1640 + 10% FBS
OVCAR4	ATCC	RPMI 1640 + 10% FBS
OVKATE	Japanese Collection of Research Bioresources Cell Bank	RPMI 1640 + 10% FBS
COV318	European Collection of Authenticated Cell Cultures	DMEM + 10% FBS
OVSAHO	Gottfried Konecny (UCLA)	RPMI 1640 + 10% FBS
CAOV3	ATCC	DMEM + 10% FBS
OVCAR8	ATCC	RPMI 1640 + 10% FBS

Supplementary Table 3. Materials used in these studies

Chemicals	Company	Cat number
Decitabine	Tocris	2624
SGI-110 (Guadecitabine)	Adooq bioscience	A12744

Antibody	Company	Cat number	Species	Methods/Dilution
LINE-1 ORF1p	EMD Millipore	MABC1152	M	WB 1:1000<br>IF 1:500
Beta actin	Cell signaling	#3700	M	WB 1:1000
HE4	abcam	ab200828	R	WB 1:1000
CA125	BIORAD	VMA00070	M	WB 1:1000
GAPDH	Cell signaling	#2118	R	WB 1:1000

Supplementary Table 4.

Sample group	Ratio Light to Heavy	Average	Standard deviation	CV	t-test	t-comparison
1 - Heavy peptide	0	0.0001	0.0001	100.0%	0.005785	Group 1 to Group 2
1 - Heavy peptide	0.0002					
1 - Heavy peptide	0.0001					
2 - Ab + heavy peptide + buffer	0.0005	0.000667	0.000153	22.9%	0.008821	Group 2 to Group 3
2 - Ab + heavy peptide + buffer	0.0008					
2 - Ab + heavy peptide + buffer	0.0007					
3 - Ab + heavy peptide + control plasma	0.0014	0.001567	0.000289	18.4%	0.004143*	Group 3 to Group 4
3 - Ab + heavy peptide + control plasma	0.0019					
3 - Ab + heavy peptide + control plasma	0.0014					
4 - Ab + heavy peptide + patient plasma	0.0058	0.006933	0.00155	22.4%	0.001593*	Group 4 to Group 1
4 - Ab + heavy peptide + patient plasma	0.0087					
4 - Ab + heavy peptide + patient plasma	0.0063					
